# Correlation between RNA N6-methyladenosine and ferroptosis in cancer: current status and prospects

**DOI:** 10.3389/fcell.2024.1252064

**Published:** 2024-03-13

**Authors:** Qianzi Liu, Linxi Lv, Xueding Cai, Jiandong Zhu, Jifa Li, Lehe Yang, Xiaona Xie, Chengguang Zhao, Haiyang Zhao

**Affiliations:** ^1^ Institute of Life Sciences, Biomedical Collaborative Innovation Center of Zhejiang Province, Wenzhou University, Wenzhou, Zhejiang, China; ^2^ The First Affiliated Hospital of Wenzhou Medical University, Wenzhou, China; ^3^ Affiliated Yueqing Hospital, Wenzhou Medical University, Wenzhou, Zhejiang, China

**Keywords:** N6-methyladenosine, ferroptosis, cancer, correlation, regulation

## Abstract

N^6^-methyladenosine (m^6^A) is the most abundant chemical modification in eukaryotic cells. It is a post-transcriptional modification of mRNA, a dynamic reversible process catalyzed by methyltransferase, demethylase, and binding proteins. Ferroptosis, a unique iron-dependent cell death, is regulated by various cell metabolic events, including many disease-related signaling pathways. And different ferroptosis inducers or inhibitors have been identified that can induce or inhibit the onset of ferroptosis through various targets and mechanisms. They have potential clinical value in the treatment of diverse diseases. Until now, it has been shown that in several cancer diseases m^6^A can be involved in the regulation of ferroptosis, which can impact subsequent treatment. This paper focuses on the concept, function, and biological role of m^6^A methylation modification and the interaction between m^6^A and ferroptosis, to provide new therapeutic strategies for treating malignant diseases and protecting the organism by targeting m^6^A to regulate ferroptosis.

## Introduction

N6-methyladenosine (m^6^A) is a frequent post-transcriptional modification in many eukaryotic mRNAs, and was first reported in 1974 (Fu et al., 2014). This modification is a highly dynamic and reversible process involving the installation of methyltransferases called “writers,” the removal of demethylases called “erasers,” and the recognition of binding proteins called “readers.” m^6^A can regulate mRNA processing events such as translation, export, selective splicing, and stability. A growing body of evidence suggests that m^6^A modification is involved in carcinogenesis. For example, it regulates cell metastasis, proliferation, stem cell differentiation, and homeostasis in cancer and represents a promising biomarker for cancer detection (Qing et al., 2021; Zhao et al., 2021).

Ferroptosis, a novel form of cell death discovered recently, plays a vital role in clearing malignant cells. It is caused by iron-dependent lipid peroxidation and excessive accumulation of reactive oxygen species. It is distinguished from apoptosis, necrosis, pyroptosis, and autophagy, mainly characterized by lipid peroxidation, glutathione peroxidase 4 (GPX4) deficiency, and mitochondrial membrane density concentration (Chen et al., 2021b). Ferroptosis has important implications for diseases such as cancer, aging, neurodegenerative diseases, and ischemia-reperfusion injury. The related signaling pathways have been identified as therapeutic targets in several investigations (Sun et al., 2019).

Recently, many researchers have focused on ferroptosis, investigating whether changes in the critical m^6^A enzyme can directly or indirectly induce ferroptosis, resulting in various disease problems. In this review, we explore how m^6^A methylation is involved in the initiation and progression of ferroptosis and the prospects of using m^6^A methylation as a new diagnostic biomarker and therapeutic target for disease treatment.

## m^6^A modification and its regulators

**FIGURE 1:**
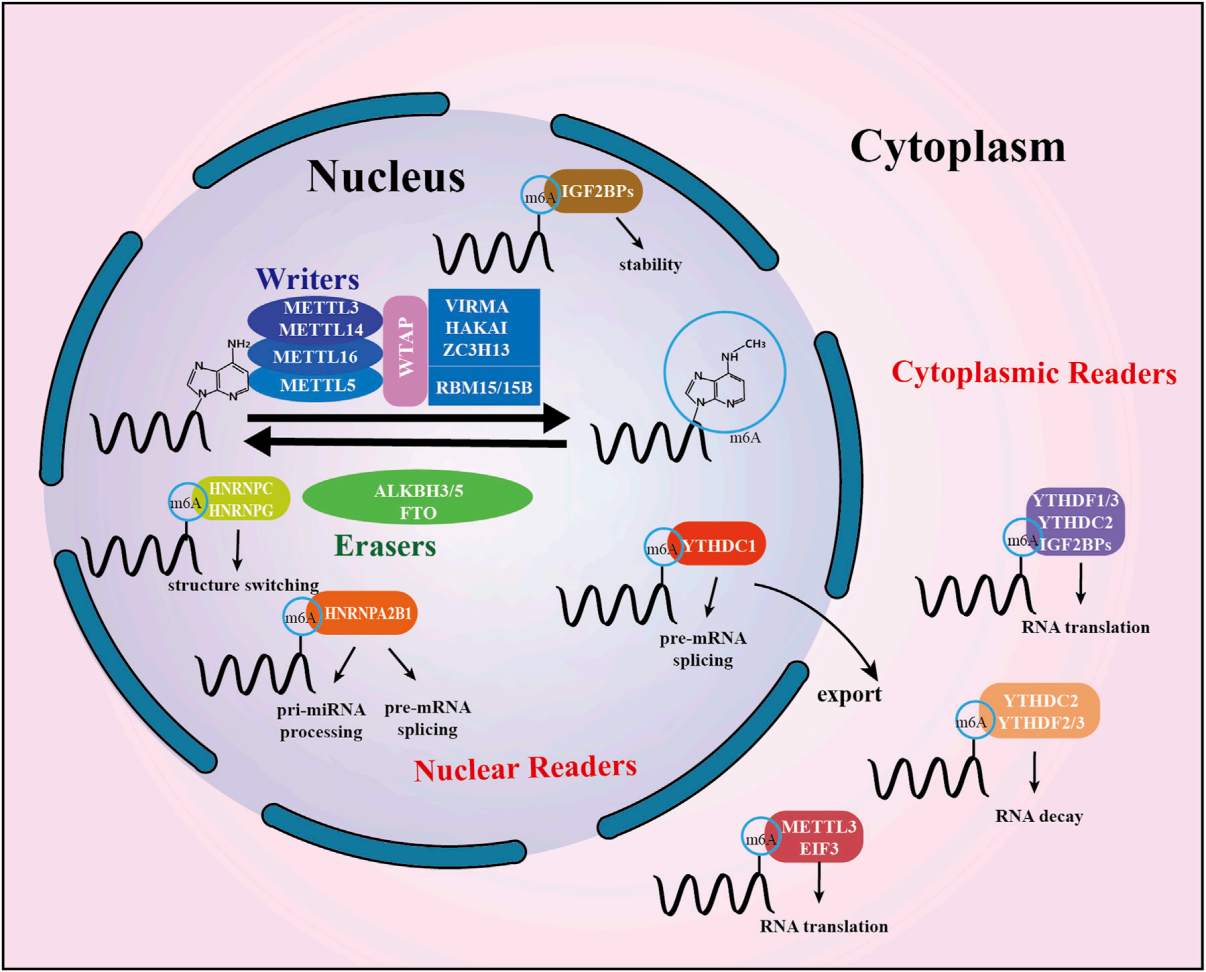
Summary of m^6^A modification machinery. The m^6^A methyltransferase complex composed of METTL3, METTL14, and WTAP, probably also of VIRMA and RBM15, serves as m^6^A “writer”, demethylases (FTO and ALKBH5) serve as m^6^A “erasers”. A set of m^6^A-binding proteins serve as m^6^A “readers” that determine the fate of target m^6^A-modified mRNA transcripts. Mature RNAs modified by m^6^A in the nucleus are recognized by readers, which subsequently mediate subcellular localization. In the cytoplasm, m^6^A is identified by cytoplasmic readers. It modulates RNA stability, translation, and binding capacity.

The m^6^A “writers,” “erasers,” and “readers” are proteins that can add, remove, or recognize m^6^A modification sites, altering critical biological processes in the process. The members of each class of regulators work together in a coordinated manner to maintain a stable balance of m^6^A levels in the cell. The specific details are described below, and a summary is presented in Figure 1.

### Methyltransferase: m^6^A writers

The methylation of m^6^A is catalyzed by a multicomponent methyltransferase complex, which is mediated by the methyltransferase complex (MTC). The first characteristic component of the m^6^A methyltransferase complex is METTL3, an S-adenosyl-methionine (SAM) binding protein (Bokar et al., 1997). Another active component of the m^6^A methyltransferase complex, METTL14, co-localizes with METTL3 in the nuclear speckle and forms a stable heterogeneous complex in a 1:1 ratio with METTL3 (Liu et al., 2014). WTAP (Wilms tumor 1-associated protein) is the complex’s third key component (Wang et al., 2016). RBM15 and its analog RBM15B were later discovered to be members of a complex that facilitates their recruitment to specific sites on RNA molecules (Patil et al., 2016). Recently, researchers have identified vir-like m^6^A methyltransferase-associated proteins VIRMA (also known as KIAA1429), HAKAI and zinc finger protein 13 (ZC3H13) to interact with other components of the complex to form a large multi-component complex (Yue et al., 2018). In addition to this, METTL16, a homolog of METTL3, is thought to be a regulator of cellular SAM levels (Zhang et al., 2019). It can catalyze m^6^A modification in small nuclear RNAs, U6 snRNAs, and other non-coding RNAs (lncRNAs) (Warda et al., 2017). METTL5 has a similar function (van Tran et al., 2019) (Figure 1).

### Demethylases: m^6^A erasers

In 2011, the first m^6^A demethylase, Fat mass and obesity-associated protein (FTO) were identified, indicating that m^6^A RNA methylation is reversible and dynamic (Jia et al., 2011). A second RNA demethylase, α-ketoglutarate-dependent dioxygenase homolog 5 (ALKBH5), which is predominantly expressed in the testis, was soon discovered in 2013 (Zheng et al., 2013; Ueda et al., 2017). The recently discovered demethylase ALKBH3, which appears to demethylate tRNA preferentially, is also commonly thought to be a DNA repair enzyme (Beharry et al., 2016) (Figure 1).

### Methylation recognition protein: m^6^A readers

The m^6^A methylation recognition protein activates downstream signaling pathways by selectively recognizing bases where m^6^A modifications occur, which regulates mRNA export, translation, metabolism, and stability. The m^6^A methylation recognition proteins can be broadly classified according to their functions: YTHDF2, YTHDF3, and YTHDC2, which promote mRNA degradation; IGF2BP1/2/3, which maintain mRNA stability; and YTHDF1, YTHDF3, YTHDC2 and IGF2BP1/2/3, which promote translation of target mRNAs(Huang et al., 2018); YTHDC1, on the other hand, may affect mRNA splicing and export from the nucleus (Wang et al., 2014). Members of the heterogeneous nuclear ribonucleoprotein (HNRNP) family (HNRNPA2B1, HNRNPC and HNRNPG) are potential m^6^A methylation recognition proteins that play a crucial role in regulating the processing and maturation of RNA substrates and gene expression (Lin et al., 2016). In addition, METTL3 in the cytoplasm can act as a reader protein and promote the translation of certain specific cellular mRNA types (Alarcon et al., 2015). Eukaryotic initiation factor 3 (EIF3), proline-rich coiled-coil protein 2A (PRRC2A), and other recognition proteins have also been reported (Wu et al., 2019) (Figure 1).

### Biological functions of m^6^A modifications

As a widely distributed RNA modification with many recognition proteins, m^6^A mediates various biological functions involved in the regulation of mRNAs, the direction of differentiation of stem cells, the regulation of development, the development of neurological diseases and the progression of tumors.

RNA m^6^A modification appears to be transcription-independent. However, a recent study revealed that chromosome-associated regulatory RNAs (carRNAs), can be modified by m^6^A to cause carRNA attenuation via YTHDC1, influencing chromatin state and downstream transcription (Liu et al., 2020). In mammals, m^6^A modifications can regulate the pathway of pre-mRNA splicing. For example, the m^6^A recognition protein HNRNPG may interact with RNA polymerase II and nascent pre-mRNA co-transcription using the Arg-Gly-Gly motif to control selective splicing (Zhou et al., 2019). Additionally, YTHDC1 can direct pre-mRNA splicing factors to bind to certain mRNAs and regulate mRNA splicing (Xiao et al., 2016). There is also evidence that m^6^A can alter RNA structure and thus affect RNA-protein interactions in cells. In addition, it was found that m^6^A is involved in regulating biological development and plays a vital role in sex determination (Haussmann et al., 2016), spermatogenesis (Xu et al., 2017), epithelial cell differentiation (Wang et al., 2017) and other functions (Huang et al., 2020).

According to a study, lung cancer patient’s circulating tumor cells (CTCs) of considerably increased the m^6^A modification. This suggests that upregulating the m^6^A RNA methylation in CTCs may assist monitor and preventing tumor spread (Huang et al., 2016). In addition, R-2HG exhibited anti-leukemic effects as it increased m^6^A modification in sensitive cells by inhibiting FTO proteins (Su et al., 2018). Not coincidentally, the modification of m^6^A regulators in cancer has also been reported. Investigators found that METTL3 was modified by SUMO1, and significantly inhibited its m^6^A methyltransferase activity, resulting in reduced m^6^A levels in mRNA. These results suggest that modifying m^6^A regulators may be a novel molecular mechanism for regulating m^6^A methylation (Du et al., 2018).

## Ferroptosis

**FIGURE 2:**
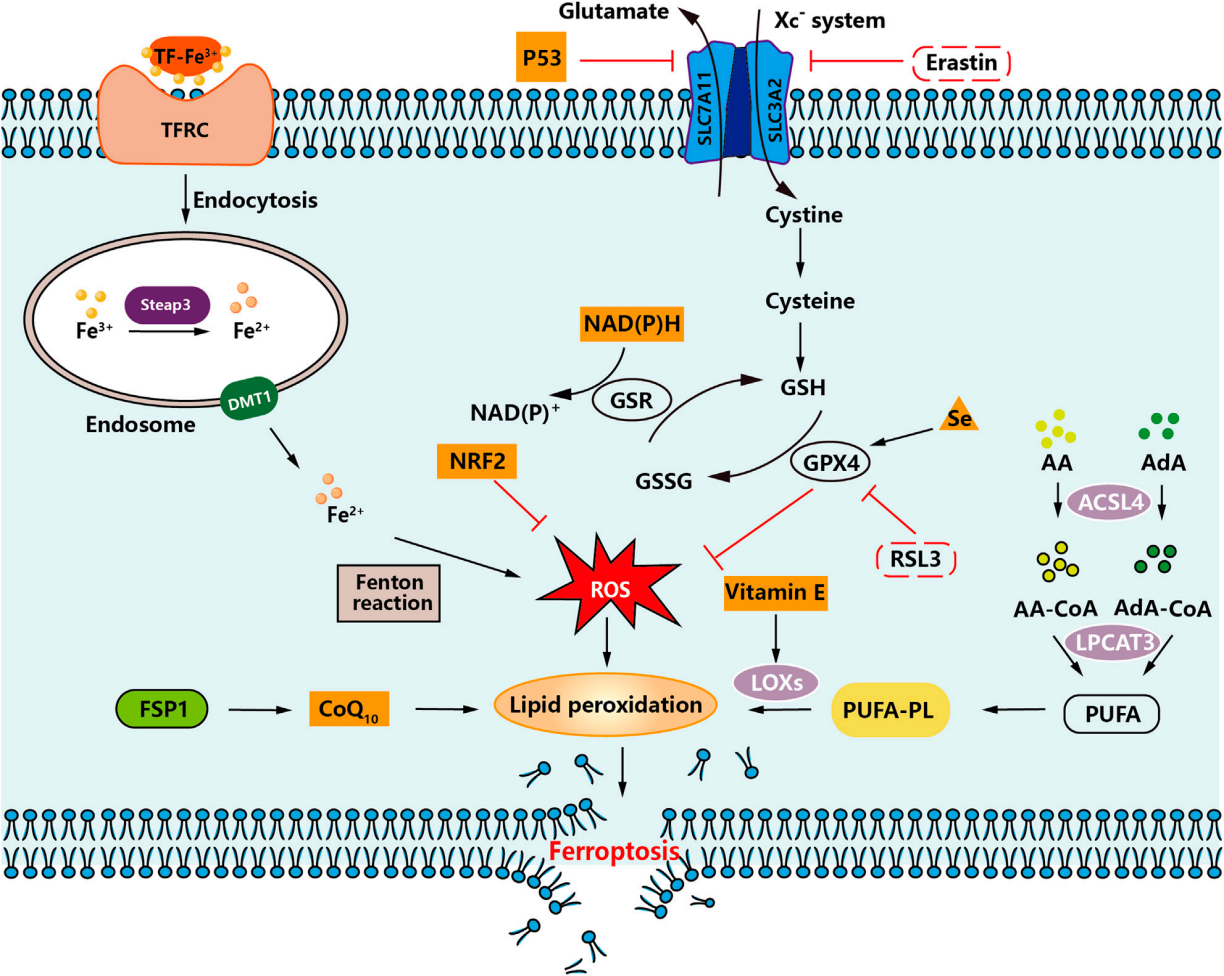
An overview of ferroptosis. An illustration demonstrating how intracellular and intercellular signaling events can influence ferroptosis by regulating cellular metabolism and ROS levels.

Ferroptosis is an iron-dependent, unique kind of programmed cell death separate from apoptosis, cell necrosis, and cell autophagy initially postulated by Dr. Brent R. Stockwell at Columbia University in 2012 (Dixon et al., 2012; Yang et al., 2014). The specific flow chart is shown in Figure 2.

The following features mainly characterize ferroptosis: (i) The accumulation of significant levels of iron ions, lipid peroxidation, increased ROS, and alterations in specific genes that regulate iron homeostasis and lipid peroxidation metabolism, are all associated with cell death. (ⅱ) In the fine structure of the cell, smaller than average mitochondria will appear, and the mitochondrial membrane will be wrinkled. In contrast, the mitochondrial cristae will be reduced or disappear, and the outer membrane will be broken, but the morphological changes in the nucleus are not apparent (Cosialls et al., 2021).

### Regulation of ferroptosis

Ferroptosis is a regulated form of cell death characterized by accumulating iron-mediated lipid peroxidation of polyunsaturated fatty acid (PUFA) to lethal levels. Sensitivity to ferroptosis is closely related to many biological processes, including amino acid, iron, and polyunsaturated fatty acid metabolism (Stockwell et al., 2017). Current studies have shown that the accumulation of PUFA oxide is a marker of ferroptosis. Its accumulation process mainly involves deoxygenation inhibition (mainly GPX4 and ferroptosis suppressor protein 1 (FSP1)) and enhanced peroxidation catalyzed by iron and a series of enzymes. A detailed diagram is shown in Figure 2.

Among the amino acid metabolism pathways, the regulatory systems involved in ferroptosis are mainly the Xc^−^ system (SLC7A11/SLC3A2 complex) and the lipid repair enzyme glutathione peroxidase 4 (GPX4) system (Ursini and Maiorino, 2020). When the Xc^−^ system is inhibited, cystine uptake decreases and so does the amount of glutathione (GSH), leading to the accumulation of lipid radicals. After GPX4 inactivation, lipid radicals are converted to toxic lipid peroxides catalyzed by iron, which leads to the rupture of PUFA of cell membrane lipids and induces ferroptosis (Doll and Conrad, 2017). Therefore, when the cystine transport protein (Erastin) is inhibited, intracellular GSH is depleted, which eventually leads to the inactivation of GPX4, leading to the accumulation of lipid peroxidation, which can induce cell death. Inhibition of GPX4 enzymes, such as RSL3, can also directly contribute to this effect. Therefore, inhibition of Xc^−^ system, insufficient or depleted GSH synthesis, and inactivation of GPX4 all lead to the accumulation of lipid peroxides in the cell, and ultimately to ferroptosis (Cao et al., 2022).

In the lipid metabolism pathway, arachidonic acid (AA) and adrenaline (AdA) can be esterified by acyl-CoA synthetase long-chain family member 4 (ACSL4) to lipid coenzyme a derivatives, and then recombinant lysophosphatidyl-choline acyltransferase 3 (LPCAT3) promotes the incorporation of PUFA into phospholipids to form polyunsaturated fatty acid phospholipids (PUFA-PL) (Yang et al., 2016; Doll et al., 2017). PUFA-PLs are susceptible to oxidation triggered by free radicals mediated by lipoxygenases (LOXs). This oxidation eventually disrupts the lipid bilayer and affects membrane function, thereby promoting ferroptosis (Wu et al., 2019). Thus, activation of ACSL4, LPCAT3, and LOXs leads to excessive lipid peroxidation, resulting in the development of ferroptosis.

Among the pathways associated with iron metabolism, Fe^3+^ enters the blood and binds to transferrin (TF) to form TF-Fe^3+^, which binds to the transferrin receptor (TFRC) on the cell surface. The intracellular metal reductase STEAP3 reduces Fe^3+^ to Fe^2+^ and then releases Fe^2+^ into the dynamic iron pool of the cytoplasm via divalent metal transporter protein 1 (DMT1) (Gao et al., 2015). Under pathological conditions, Fe^2+^ accumulates in the cell. It undergoes the Fenton reaction, generating a large amount of ROS, which will generate lipid peroxides by a series of peroxidation reactions with PUFA on the cell membrane, leading to the destruction of the cell membrane structure and eventually causing ferroptosis (Yu et al., 2019).

Other factors that affect ferroptosis sensitivity include coenzyme Q10 (CoQ10) (Bersuker et al., 2019), reduced coenzyme II (Nicotinamide Adenine Dinucleotide phosphate, NADPH) (Guo et al., 2020), selenium (Alim et al., 2019), p53 (Jiang et al., 2015)., nuclear factor E2-related factor 2 (NRF2) (Sun et al., 2016), and Vitamin E (Imai et al., 2017).

### Biological functions of ferroptosis

Susceptibility to ferroptosis is tied to many biological processes, and excess iron can lead to tissue damage and an increased risk of cancer. It has also been associated with pathological cell death associated with mammalian neurodegenerative diseases (such as Alzheimer’s disease, Huntington’s chorea and Parkinson’s syndrome), stroke, cerebral hemorrhage, traumatic brain injury, local ischemia-reperfusion injury and renal failure (Jiang et al., 2021). In addition, ferroptosis is a potential weapon against the development of many cancers, such as colorectal (Liu et al., 2022b), pancreatic (Yamaguchi et al., 2018), gastric (Zhang et al., 2020), lung (Wang et al., 2019), liver (Capelletti et al., 2020), renal cell (Yang et al., 2019) and ovarian (Wang et al., 2021) cancers.

Compared with normal cells, cancer cells are more metabolically active, with dysfunctional mitochondria and more ROS accumulation, which increases the sensitivity of cancer cells to ferroptosis. Tumors are more sensitive to ferroptosis induction, namely, when they undergo epithelial-mesenchymal transition or when they acquire “stemness” and become tumor stem cells (Zhang et al., 2018). In brief, induction of ferroptosis may not only be a new means of inhibiting tumor cell growth, but also provides a new promising pathway for reversing tumor drug resistance.

Meanwhile, several studies have demonstrated the role of ferroptosis in neurodegenerative and cardiovascular diseases, suggesting the therapeutic potential of ferroptosis inhibition. Iron chelators have been tested in different experimental systems for stroke and neurodegeneration (Hambright et al., 2017). Elevated iron levels have been detected in patients with aging and degenerative diseases such as Parkinson’s disease (PD), Huntington’s disease and Alzheimer’s disease (AD) (Buijs et al., 2017). Multiple lines of evidence suggest that the GSH-GPX4 antioxidant pathway is significantly abnormal in both AD and PD patients and that using antioxidant drugs may alleviate symptoms of AD and PD pathology (Yan et al., 2021).

## m^6^A modification induces ferroptosis pathway

**FIGURE 3:**
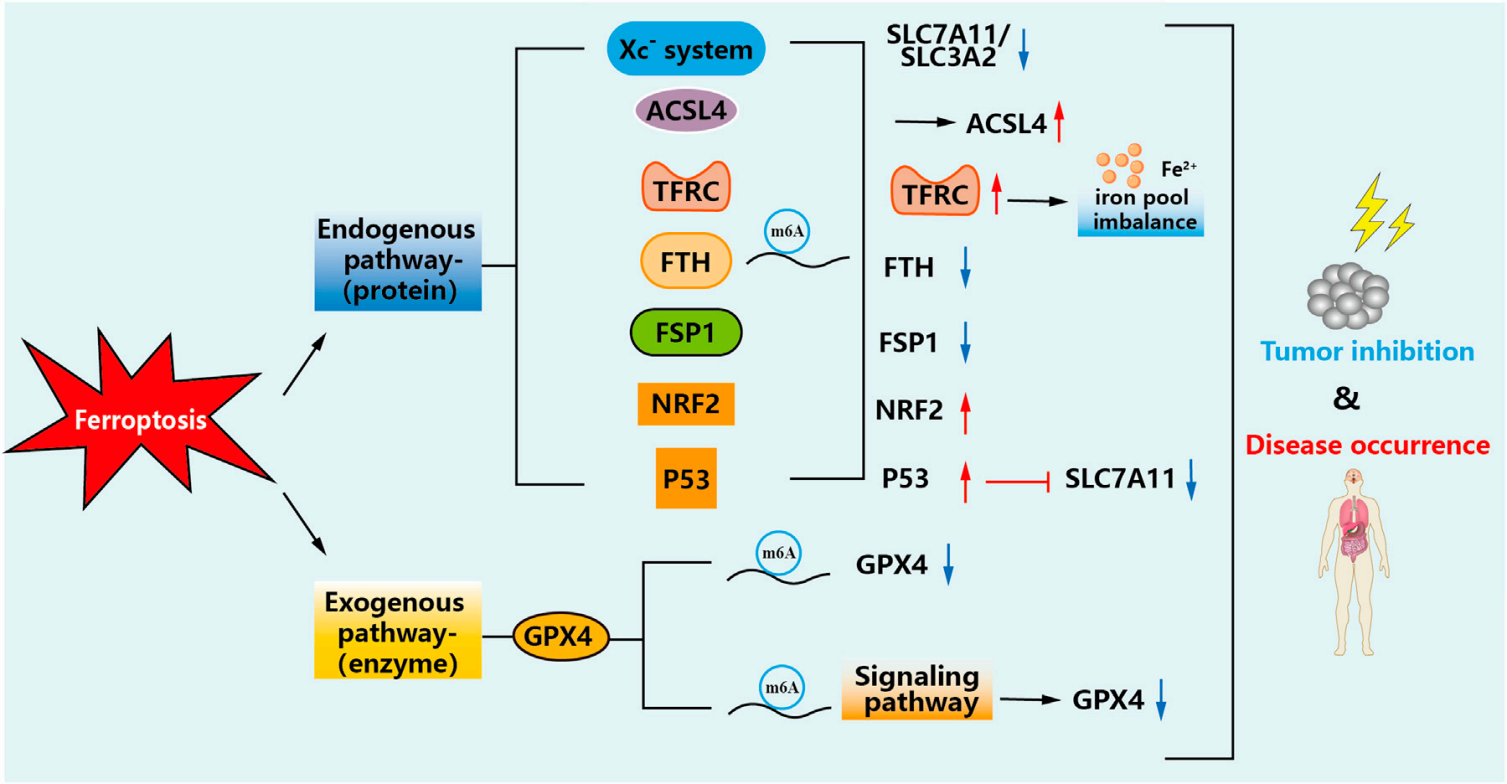
Mechanisms of m^6^A-modified ferroptosis pathways. m^6^A modification can change the metabolic course of ferroptosis in cells, regulate the changes of these factors, increase lipid peroxidation and iron accumulation in the tumor cell microenvironment, and thus inhibit the occurrence and development of tumors or reduce damage to the body.

In recent studies, multiple iron metabolism-related pathways have been found to play an essential role in the progression of tumor development, which may provide ideas for developing new therapies. However, two main pathways affect ferroptosis: exogenous (transporter protein-dependent) and endogenous (enzyme-regulated) pathways, and modifications of m^6^A are present in these cases (Chen et al., 2021a). The details are shown in Figure 3.

### Exogenous (transporter protein-dependent) pathway

The exogenous pathway enables initiation by inhibition of cell membrane transport proteins such as cystine/glutamate reverse transporter proteins (Xc^−^ system), or activation of iron transport proteins. Increasing iron uptake, decreasing iron storage and limiting iron efflux lead to increased iron accumulation, which then promotes ferroptosis through a series of signaling pathways.

In a recent study, high expression of YTHDC2 was found to induce ferroptosis in lung adenocarcinoma (LUAD) cells. SLC7A11 mRNA is modified by m^6^A and binds to YTHDC2 to inhibit the antioxidant function of LUAD cells by accelerating the decay of SLC7A11 mRNA (Ma et al., 2021a). Another subunit of the Xc^−^ system, SLC3A2, is equally essential for YTHDC2-induced ferroptosis. HOXA13 acts as a transcription factor to stimulate the expression of SLC3A2. Thus, YTHDC2 inhibits SLC3A2 by repressing HOXA13 indirectly through m^6^A (Ma et al., 2021b). Similarly, METTL3-mediated m^6^A modification stabilizes SLC7A11 mRNA and promotes its translation, thereby promoting LUAD cell proliferation and inhibiting ferroptosis. This is because the m^6^A reader YTHDF1 is recruited by METTL3 to enhance the modification of SLC7A11 m^6^A (Xu et al., 2022). Not coincidentally, in the modification of SLC7A11 by METTL3, IGF2BP1 promoted the stability of SLC7A11 mRNA and upregulated its expression by inhibiting the process of demethylation thereby suppressing the sensitivity of hepatoblastoma to ferroptosis before leading to disease progression (Liu et al., 2022a). Exogenous overexpression of NKAP protects glioblastoma cells from ferroptosis by positively regulating SLC7A11, promoting cell resistance to ferroptosis inducers (Alarcon et al., 2015).

As an m^6^A demethylase, FTO downregulates SLC7A11 through m^6^A demethylation, regulating ferroptosis in papillary thyroid carcinoma (PTC), thereby inhibiting the development of PTC (Ji et al., 2022). FTO removes m^6^A modification of OTUB1 transcripts and promotes the expression of OTUB1. By stabilizing the interaction between OTUB1 and SLC7A11, FTO inhibits radiation-induced cell ferroptosis and ultimately triggers radiotherapy resistance in nasopharyngeal carcinoma (Huang et al., 2023). ALKBH5 eliminated the m^6^A modification on SLC7A11 mRNA, reduced the stability of SLC7A11 mRNA, and further reduced the expression of SLC7A11, thus promoting ferroptosis in CRC cells (Luo et al., 2023). Using drugs to induce ferroptosis is a popular strategy. Curdione activates ferroptosis by enhancing MELL14 and the methylation of SLC7A11 mRNA and HOXA13 mRNA, reducing the stability of the reading protein YTHDF2, leading to the expression of SLC3A2. These results provide references or new ideas for the study of ferroptosis in colorectal cancer (Wang et al., 2023). Inhibition of METTL14 is triggered in an HIF-1α-dependent manner under hypoxia conditions, and SLC7A11 was found to be a direct target of METTL14. METTL14 induces m^6^A modification of SLC7A11 mRNA at 5′UTR, and its degradation depends on a YTHDF2-dependent pathway, which can effectively eliminate ferroptosis in HCC cells (Fan et al., 2021). At the same time, a potential m^6^A modification site on SLC7A11 mRNA was also found in another study, and YTHDF2 can bind to SLC7A11 mRNA in an m^6^A dependent manner. YTHDF2 promotes the degradation of SLC7A11 mRNA, thereby reducing its mRNA stability. Therefore, YTHDF2 accelerates the ferroptosis of endothelial cells in cerebrovascular atherosclerosis (Li et al., 2023). KIAA1429, a key component of the m^6^A methyltransferase complex, targets SLC7A11 during ferroptosis in Hepatocellular carcinoma (HCC) cells. It protects HCC cells against ferroptosis and provides a new insight into abnormally regulated external transcriptomics in the HCC context (Wang et al., 2023). The resulting discovery of a vital role for m^6^A modification in Xc^−^system-mediated ferroptosis provides a potential strategy for treating the disease by blocking the m^6^A- Xc^−^ system axis.

ACSL4 is a member of the long chain family of acyl-CoA synthetase proteins and is one of the core factors of lipid peroxidation during ferroptosis after myocardial I/R injury. Inhibition of ACSL4 can prevent ferroptosis and reduce myocardial and kidney I/R damage (Qiu et al., 2023). The LPCAT3 protein can also interact with ACSL4, and its upregulation inhibits the expression of ACSL4. *In vitro* models, ACSL4 was identified as a target for LPCAT3 to inhibit mitochondrial damage. Methylation regulation of LPCAT3 improves osteoarthritis by regulating ACSL4 to inhibit chondrocyte ferroptosis (Habaxi et al., 2024).

The transferrin receptor (TFRC) is essential for the uptake of the transferrin - iron complex into cells. It has been shown that in head and neck squamous cell carcinomas (HNSCCs), the YTH structural domain of the m^6^A reader YTHDF1 interacts with the 3′UTR and 5′UTR of TFRC mRNA. It positively regulates the translation of m^6^A-modified TFRC mRNA. The high expression of YTHDF1 may increase iron metabolism by promoting the accumulation of intratumoral iron (Jung et al., 2019). From a therapeutic perspective, targeting YTHDF1 and TFRC-mediated iron metabolism to promote iron homeostasis imbalance may be a promising strategy for treating HNSCC. Meanwhile, upregulation of METTL3 lactation enhanced the stability and expression level of METTL3 protein in hemin-treated PC12 cells. After METTL3 silencing, the occurrence of ferroptosis can be inhibited by regulating the m^6^A level of TFRC mRNA, which is a method to treat cerebral hemorrhage (Zhang et al., 2023).

Ferritin (FTH) is a key regulator of ferroptosis. Downregulated YTHDF1 inhibited cell proliferation, migration and invasion. FTH has been identified as a key target for YTHDF1. In addition, overexpression in YTHDF1 deficient cells partially restored the inhibition. Thus, YTHDF1 upregulation promotes lung cancer by accelerating ferritin translation in an m^6^A-dependent manner (Diao et al., 2023). Other studies have shown that LncRNA CACNA1G-AS1 can upregulate the expression of FTH1 through the IGF2BP1 axis, thereby inhibiting ferroptosis by regulating FTH phagocytosis, and ultimately promoting the proliferation and migration of ovarian cancer cells (Jin et al., 2023).

FSP1 is a necessary ferroptosis inhibitor. High levels of miR-4443 inhibit cisplatin induced tumor death by reducing the expression level of METTL3 and increasing the level of FSP1, suggesting that miR-4443 plays an important role in cisplatin resistance in NSCLC through the METTL3/FSP1 mediated ferroptosis (Song et al., 2021). Downregulation of YTHDC1 promotes lung tumor progression and leads to ferroptosis resistance through m^6^A mediated upregulation of FSP1 protein levels, providing a treatment option for lung cancer with high YTHDC1 levels associated with ferroptosis (Yuan et al., 2023). The upregulation of METTL3 and FSP1 induced by fear stress increases the m^6^A level of glioma tumor tissue, providing a new understanding of glioma development by inhibiting the tumor progression caused by ferroptosis (Bu et al., 2023).

NRF2 plays a crucial role in the regulation of cellular antioxidant molecules. It has been reported that there is a significant m^6^A modification site on the 3′UTR, and WTAP can install its methylation. In addition, YTHDF1 identifies the m^6^A site on NRF2 mRNA and enhances its mRNA stability, thereby accelerating the progression of bladder cancer malignancies (Wang et al., 2023). In another study, the action mechanism and detailed mechanism of SNAI3-AS1/SND1/NRF2 in glioma ferroptosis were elucidated. lncRNA SNAI3-AS1 interfered with SND1’s m^6^A recognition of NRF2 mRNA, thereby reducing the stability of NRF2 mRNA and promoting the occurrence of ferroptosis (Zheng et al., 2023). NRF2 has also been found to be a target of FTO, which demethylates m^6^A in NRF2 mRNA, thereby impairs the stability of NRF2 mRNA. Therefore, the results of this study illustrate the important role of FTO in Parkinson’s disease (PD) through the ferroptosis (Pang et al., 2024).

p53 can inhibit cystine uptake by down-regulating the expression of SLC7A11, thereby inducing ferroptosis. Erianin significantly increased the m^6^A modification level of the 3′UTR of ALOX12 and P53 mRNA in Human renal cancer stem cells (HuRCSCs). By promoting modification of m^6^A, kidney cancer stem cells can be induced by ferroptosis, and finally have therapeutic effect (Shen et al., 2023). Silencing KIAA1429 promoted erastin -induced NSCLC cell ferroptosis, activated the p53 signaling pathway, and inhibited its proliferation, migration, and invasion. This also suggests that m^6^A may be a molecular target for a promising therapeutic strategy for the treatment of NSCLC (Wu et al., 2023).

### Endogenous (enzyme-regulated) pathways

The endogenous pathway is activated by blocking intracellular antioxidant enzymes (e.g., GPX4). Lipid peroxide accumulation is a marker of ferroptosis, GPX4 reduces cytotoxic lipid peroxides (L-OOH) to the corresponding alcohol (L-OH), and inhibition of GPX4 activity leads to the accumulation of cell membrane lipid peroxides (Conrad and Pratt, 2019). Studies have shown that FTO can directly regulate the m^6^A modification of GPX4 mRNA at site 193, while YTHDF2 regulates AKT inhibitation-induced ferroptosis by recognizing and degrading the m^6^A methylation modification site on GPX4 mRNA (Zhang et al., 2023). RUNX1-IT1 can directly bind to IGF2BP1, resulting in IGF2BP1 occupying more of GPX4 mRNA and increasing the stability of GPX4 mRNA. Targeting the previously unappreciated RUNX1-IT1/IGF2BP1/GPX4 regulatory axis may be a promising treatment for breast cancer patients (Wang et al., 2023). METTL16 epigenetically enhances GPX4 expression through m^6^A modification and promotes breast cancer progression by inhibiting ferroptosis (Ye et al., 2023). By demethylating the m^6^A modification of ACSL3 and GPX4 mRNA, FTO reduces its stability and inhibits the expression of ACSL3 and GPX4. Ferroptosis activation of high levels of FTO in Oral squamous cell carcinoma (OSCC) may be a potential therapeutic target (Wang et al., 2023) found that METTL3 was involved in high glucose and palmitic acid (HGPA)-induced osteoporosis through upregulation of the ASK1/p38 signaling pathway, and that activation of the ASK1/p38 pathway was associated with ferroptosis induction. This study suggests that high glucose and high fat (HGHF) induces ferroptosis in diabetic osteoporosis by activating the METTL3/ASK1-p38 pathway (Lin et al., 2022).

## m^6^A modification regulates the occurrence of ferroptosis

**FIGURE 4:**
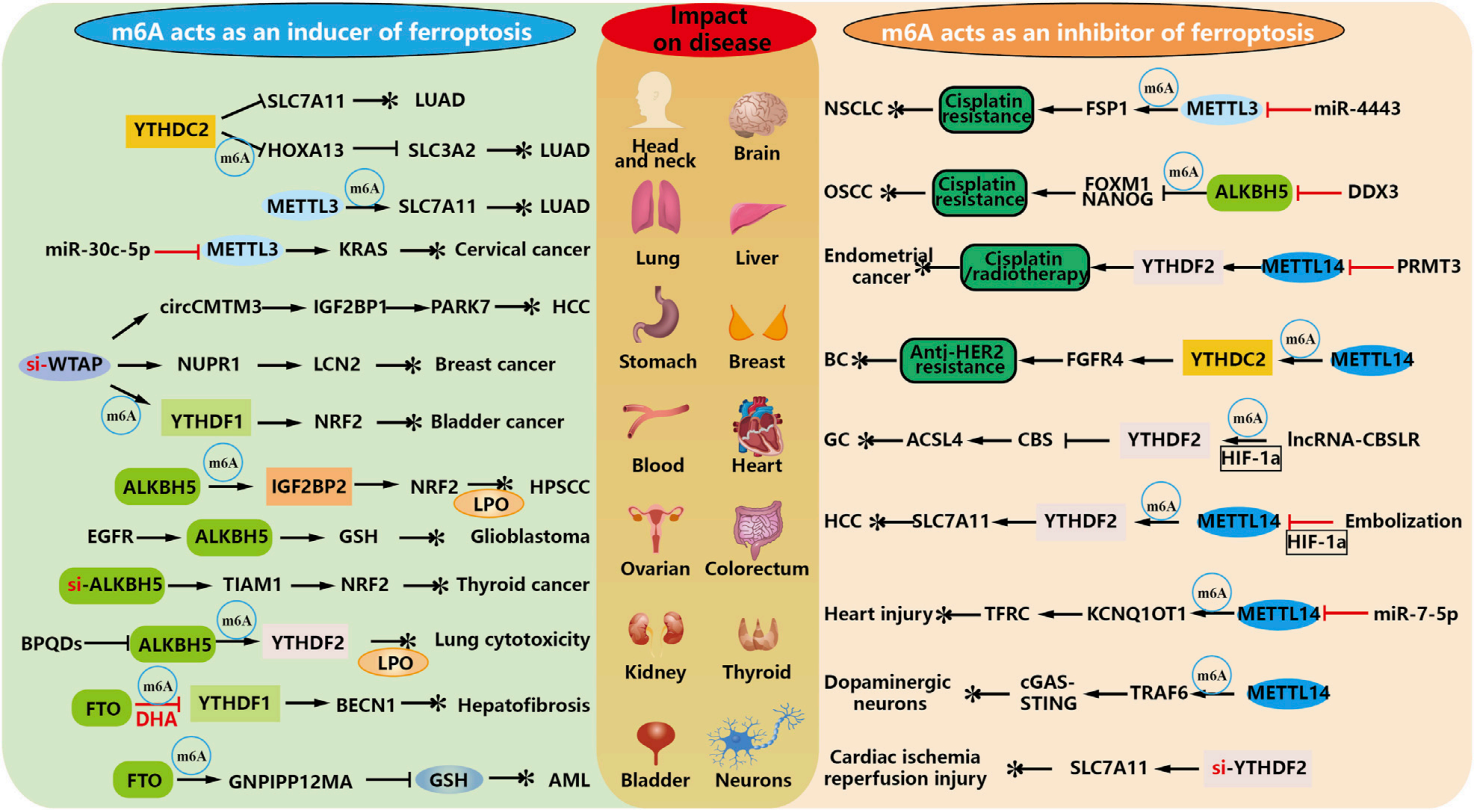
m^6^A modification regulates the inhibition and occurrence of ferroptosis. As an inducer of modified regulated ferroptosis, m^6^A elucidated that exogenous drugs or high expression of m^6^A in tumors promoted ferroptosis and thus played an anticancer role. At the same time, m^6^A methylation regulates RNA, which can affect the drug resistance of some diseases, thus producing essential side effects for its treatment.

Ferroptosis inducers have been shown to enhance the antitumor effects of radiation, but ferroptosis inhibitors have been developed to attenuate radiation-induced organ damage and hematopoietic damage. Therefore, finding the linkage between m^6^A and ferroptosis in diseases where they are jointly involved, and delving into the regulatory ferroptosis pathway to maximize the clinical benefit in treatment are crucial questions that deserve further investigation. As shown in Figure 4.

### m^6^A as an inducer of ferroptosis occurrence

Tumors are the number one problem in the world, and in the treatment of oncological diseases, the most crucial purpose of m^6^A involvement in the biology of ferroptosis is to promote the occurrence of ferroptosis in tumor cells. Combined with the above description, the effect of eliminating malignant tumors can be achieved by activating the metabolic pathway of ferroptosis. For example, both subunits of the Xc^−^system in the development of lung adenocarcinoma are indispensable for YTHDC2 induced ferroptosis (Ma et al., 2021b).

In addition, METTL3-mediated m^6^A modification can stabilize SLC7A11 mRNA and promote its translation, thereby promoting the proliferation of LUAD cells and inhibiting ferroptosis. Down-regulating METTL3 can inhibit this process (Xu et al., 2022). Similarly, miR-30c-5p promotes the ferroptosis of cervical cancer by targeting the METTL3/KRAS axis, and inhibits the growth and metastasis of xenografts of cervical cancer. Targeting this process can be effectively treated (Gong et al., 2024).

WTAP-mediated m^6^A modification on circCMTM3 inhibits ferroptosis in hepatocellular carcinoma by recruiting IGF2BP1 to increase PARK7 stability (Chen et al., 2023). WTAP also can regulate NUPR1 m^6^A modification to upregulate LCN2, thereby promoting the proliferation, migration and invasion of breast cancer (Tan et al., 2023). Meanwhile, WTAP accelerates bladder cancer malignancy by targeting NRF2 through YTHDF1-m^6^A dependent ferroptosis regulation (Wang et al., 2023). Each of these pathways can play a therapeutic role by inhibiting WTAP.

One study found that ALKBH5 leads to impaired stability of NFE2L2/NRF2 mRNA and reduced expression in hypopharyngeal squamous cell carcinoma (HPSCC) through an m^6^A-IGF2BP2-dependent mechanism. Therefore, ferroptosis as an anti-cancer treatment may help treat HPSCC patients with high ALKBH5 expression (Ye et al., 2022). EGFR blocks the nuclear output of ALKBH5, increases the clearance function of m^6^A, and protects glioblastoma from ferroptosis by producing GSH. ALKBH5 inhibitors enhance the antitumor efficacy of EGFR or GSH inhibitors (Lv et al., 2023). However, ALKBH5 expression is decreased in thyroid cancer, and ferroptosis can be induced by overexpression of ALKBH5 along the m^6^A-TIAM1-NRF2/HO-1 axis, thereby inhibiting the progression of thyroid cancer, suggesting that ALKBH5 may be a potential target molecule for the treatment and diagnosis of thyroid cancer (Li et al., 2023). In contrast, in studies of nanoengineering for disease, black phosphorus quantum dots (BPQDs) significantly increased m^6^A levels and decreased levels of the demethylase ALKBH5 in lung cells. YTHDF2 recognizes these m^6^A-modified mRNAs, which leads to their decay in the cytoplasm. Ultimately, ferroptosis is triggered by biological processes including mitochondrial dysfunction, lipid peroxidation and iron overload (Ruan et al., 2021).

DHA treatment increased autophagy levels in HSCs, and upregulated m^6^A modification was essential for DHA to activate autophagy by reducing FTO (Shen et al., 2022). It ultimately plays an anti-fibrotic role by inhibiting the activation of hematopoietic stem cells (Shen et al., 2021). Self-assembled FTO inhibitor-loaded GSH bioimprinted nanocomposites (GNPIPP12MA) enhance improved GSH depletion and enhanced anti-leukemic chemo-immunotherapy. GNPIPP12MA selectively targets AML cells in the myeloid ecotone and induces ferroptosis by depleting intracellular GSH to achieve a therapeutic effect (Cao et al., 2022).

### m^6^A as an inhibitor of ferroptosis occurrence

It is believed that a significant obstacle to cancer treatment is chemotherapy resistance. Therefore, identifying new mechanisms of chemoresistance may improve clinical outcomes.

In a study of non-small cell lung cancer (NSCLC), high expression of miR-4443 was found to negatively regulate the m^6^A modification of FSP1 induced by methyltransferase METTL3 to inhibit cisplatin-induced ferroptosis, thereby conferring cisplatin resistance in NSCLC. Therefore, researchers have proposed a novel anticancer strategy to restore METTL3/FSP1-mediated ferroptosis in tumor cells (Song et al., 2021). Some investigators also found that DDX3 reduces tumor stem cell populations by inhibiting the expression of FOXM1 and NANOG. The m^6^A demethylase ALKBH5 was also directly regulated by DDX3, leading to reduced m^6^A methylation in FOXM1 and NANOG nascent transcripts, resulting in chemoresistance. In a patient-derived cell xenograft model of chemo-resistant OSCC, ketorolac restores cisplatin-mediated ferroptosis and significantly reduces tumor burden (Shriwas et al., 2020). PRMT3 inhibitor-mediated METTL14 overexpression promotes methylation through m^6^A-YTHDF2-dependent mechanism, reduces the stability of low GPX4 mRNA, increases lipid peroxidation levels, and accelerates ferroptosis. Thus, blocking PRMT3 may improve the progression of endometrial cancer after cisplatin and radiotherapy (Wang et al., 2023). Moreover, m^6^A hypermethylation resulting from METTL14 modification and YTHDC2 modulation led to the upregulation of FGFR4, which inhibited ferroptosis and conferred anti-HER2 resistance to breast cancer. Further inhibition of FGFR4 in drug-resistant cells revealed that the expression of SLC7A11 and GPX4 was significantly reduced, which promoted ferroptosis to inhibit tumorigenesis, thus restoring the sensitivity of drug-resistant breast cancer cells to anti-HER2 (Zou et al., 2022). In addition, Yang H et al. found that CBSLR, a long-stranded non-coding RNA activated by HIF-1α reverse transcription, is upregulated in gastric cancer and that inhibition of CBSLR under hypoxic conditions leads to chemoresistance. It was shown that lncRNA-CBSLR recruits YTHDF2 protein and CBS mRNA to form a CBSLR/YTHDF2/CBS complex, reducing the stability of CBS mRNA in an m^6^A-dependent manner. It protects GC from ferroptosis in the hypoxic tumor microenvironment, thus revealing a potential therapeutic target for refractory hypoxic tumors (Yang et al., 2022). Fan et al. found that METTL14 targets m^6^A methylation under normoxic conditions at the 5′UTR of SLC7A11 mRNA. m^6^A-modified SLC7A11 mRNA is recognized by YTHDF2 and then sent to the P-body for degradation. Depleting SLC7A11 leads to reduced input of cystine, cysteine and GSH accumulation, which ultimately stimulates ROS production and induces ferroptosis. In contrast, interventional embolization-induced hypoxia inhibits METTL14 in a HIF-1α-dependent manner and subsequently blocks METTL14/YTHDF2/SLC7A11/ROS axis-mediated ferroptosis, promoting HCC progression. This also suggests that targeting the HIF-1α/METTL14/YTHDF2 signaling axis may synergistically affect HCC interventions (Fan et al., 2021).

Of course, in treating malignant tumor diseases, the ultimate goal of studying the mechanisms involved in inhibiting ferroptosis by m^6^A is still to induce its occurrence to inhibit the development of tumor cells. And in the process of traumatic repair, there is also the action of chemotherapeutic drugs causing the malignant growth of normal tissues, which requires the inhibition of ferroptosis. miR-7-5p could effectively inhibit the expression of METLL14 and TFRC and selectively inhibit METTL14/KCNQ1OT1/miR-7-5p positive feedback loop-mediated ferroptosis, which could be used for the treatment of doxorubicin (DOX)-induced cardiac injury, and the approach was able to inhibit the activation of death without necessarily impairing its anticancer activity (Zhuang et al., 2021). METTL14 downregulates the expression of TRAF6 and inactivates the cGAS-STING pathway, thereby alleviating mitochondrial dysfunction and ferroptosis in dopaminergic neurons (Shao et al., 2023). The cardioprotective effect of silencing YTHDF2 is achieved by increasing the stability and expression of SLC7A11, reducing ferroptosis, and providing a new potential therapeutic target for the treatment of ischemic heart disease (Pang et al., 2023).

Taken together, these findings suggest that ferroptosis due to m^6^A modification is essential for regulating cellular homeostasis. m^6^A modification leading to ferroptosis promotes or inhibits malignant cells depending mainly on the level of m^6^A (dynamically regulated by writers and erasers), the function of downstream targets, and changes in target RNA after methylation (depending on the readers), which in turn affects lipid peroxidation or antioxidant enzymes. These findings support the identification of m^6^A regulators involved in aberrant expression in the ferroptosis pathway and as promising biomarkers to predict the prognosis of different cancers.

## Future and prospects

The m^6^A methylation is the most epistatic modality for mRNA modification in mammals and has played a vital role in various biological processes, such as tissue development and stem cell differentiation. And the critical impact of m^6^A in multiple types of cancer is being widely recognized. As a novel regulatory cell death mode different from cell necrosis, apoptosis, and autophagy, ferroptosis has promising applications in tumor therapy. In this paper, we analyze the mechanism of m^6^A in regulating the life process of ferroptosis through writers, erasers, and readers, and have specific effects on various diseases, and propose some correlations between them. It also suggests future research directions that still need to be further explored.

Although some progress has been made in identifying the relevant regulatory mechanisms, the exact link between m^6^A modification and ferroptosis under various pathological conditions remains to be further investigated. Current studies have focused on the regulatory factors of m^6^A, and the readers involved are mainly recognition proteins of the YTH structural domain; then, whether other kinds of readers are involved in the ferroptosis pathway and what role they play need to be further explored. Also, specific mechanisms in drug resistance and side effects of therapeutic approaches still need to be elucidated. m^6^A and ferroptosis-related genes are highly expressed in some cancers and may also cause other programmed cell death, which together exert anti-tumor effects. For example, in DHA-induced ferroptosis in HSCs, the interaction of m^6^A modification with BECN1 mRNA promoted the degradation of autophagic ferritin, which ultimately induced ferroptosis (Shen et al., 2022).

In a word, m^6^A, as the most common RNA modification, enhances the malignant biological behavior of some tumor cells. And tumor cells have a relatively strong sensitivity to ferroptosis, providing a new direction for m^6^A methylation modification of ferroptosis to adjuvant treat malignant tumors. Therefore, targeting m^6^A to induce ferroptosis disease may be a promising therapeutic strategy. Also, inhibition of ferroptosis after m^6^A modification also provides important guidance for studying the damage of chemotherapeutic drugs on the organism.

## Figures and Tables

**Table:** 

**AA**	Arachidonic acidc
**ACSL4**	Acyl-CoA synthetase long-chain family member 4
**AD**	Alzheimer’s disease
**AdA**	Adrenaline acid
**ALKBH5**	α-ketoglutarate-dependent dioxygenase homolog 5
**AML**	Acute myeloid leukemia
**BC**	Breast cancer
**BPQDs**	Black phosphorus quantum dots
**carRNAs**	chromosome-associated regulatory RNAs
**CoQ10**	Coenzyme Q10
**CRC**	Colorectal cancer
**CTCs**	Circulating tumor cells
**DHA**	Dihydroartemisinin
**DMT1**	Divalent metal transporter protein 1
**EIF3**	Eukaryotic translation initiation factor 3
**FSP1**	Ferroptosis suppressor protein 1
**FTO**	Fat mass and obesity-associated protein
**GC**	Gastric cancer
**GPX4**	Glutathione peroxidase 4
**GSH**	Glutathione
**GSR**	Glutathione reductase
**GSSG**	oxidized Glutathione
**HB**	Hepatoblastoma
**HCC**	Hepatocellular carcinoma
**HGPA**	High glucose and palmitic acid
**HNSCCs**	Head and neck squamous cell carcinomas
**HPSCC**	Hypopharyngeal squamous cell carcinoma
**HSCs**	Hematopoietic stem cells
**HuRCSCs**	Human renal cancer stem cells
**lncRNA**	long non-coding RNA
**I/R**	Ischemia/reperfusion
**LOXs**	Lipoxygenases
**LPCAT3**	Lysophosphatidyl-choline acyltransferase 3
**LUAD**	Lung adenocarcinoma
**m6A**	N6-methyladenosine
**SLC3A2**	Solute carrier family 3 member 2
**METTL14**	Methyltransferase-like 14
**METTL16**	Methyltransferase-like 16
**METTL3**	Methyltransferase-like 3
**METTL5**	Methyltransferase-like 5
**MTC**	Methyltransferases complex
**NADPH**	Nicotinamide Adenine Dinucleotide phosphate
**NRF2**	Nuclear factor E2-related factor 2
**NSCLC**	Non-small cell lung cancer
**OSCC**	Oral squamous cell carcinoma
**PD**	Parkinson’s disease
**PRRC2A**	Proline rich coiled-coil 2A
**PUFA**	Polyunsaturated fatty acids
**PUFA-PL**	Polyunsaturated fatty acid phospholipids
**RBM15/15B**	RNA binding motif protein 15/15B
**ROS**	Reactive oxygen species
**Se**	Selenium
**SLC7A11**	Solute carrier Family 7 member 11
**SOD**	Superoxide dismutase
**TF**	Transferrin
**TFRC**	Transferrin receptor
**VIRMA**	Vir like m6A methyltransferase associated
**WTAP**	Wilms’ tumor 1-associating protein
**ZC3H13**	Zinc Finger CCCH-Type Containing 13

## References

[B1] Alarcon C. R. Goodarzi H. Lee H. Liu X. Tavazoie S. Tavazoie S. F. (2015). HNRNPA2B1 is a mediator of m(6)a-dependent nuclear RNA processing events. Cell 162, 1299–1308. 10.1016/j.cell.2015.08.011 26321680 PMC4673968

[B2] Alim I. Caulfield J. T. Chen Y. Swarup V. Geschwind D. H. Ivanova E. (2019). Selenium drives a transcriptional adaptive Program to block ferroptosis and treat stroke. Cell 177, 1262–1279. 10.1016/j.cell.2019.03.032 31056284

[B3] Beharry A. A. Lacoste S. O'connor T. R. Kool E. T. (2016). Fluorescence monitoring of the oxidative repair of DNA alkylation damage by ALKBH3, a prostate cancer marker. J. Am. Chem. Soc. 138, 3647–3650. 10.1021/jacs.6b00986 26967262 PMC5105888

[B4] Bersuker K. Hendricks J. M. Li Z. Magtanong L. Ford B. Tang P. H. (2019). The CoQ oxidoreductase FSP1 acts parallel to GPX4 to inhibit ferroptosis. Nature 575, 688–692. 10.1038/s41586-019-1705-2 31634900 PMC6883167

[B5] Bokar J. A. Shambaugh M. E. Polayes D. Matera A. G. Rottman F. M. (1997). Purification and cDNA cloning of the AdoMet-binding subunit of the human mRNA (N6-adenosine)-methyltransferase. RNA 3, 1233–1247. 9409616 PMC1369564

[B6] Bu C. Hu S. Yu J. Li N. Gu J. Sheng Z. (2023). Fear stress promotes glioma progression through inhibition of ferroptosis by enhancing FSP1 stability. Clin. Transl. Oncol. 25, 1378–1388. 10.1007/s12094-022-03032-1 36484954

[B7] Buijs M. Doan N. T. Van Rooden S. Versluis M. J. Van Lew B. Milles J. (2017). *In vivo* assessment of iron content of the cerebral cortex in healthy aging using 7-Tesla T2*-weighted phase imaging. Neurobiol. Aging 53, 20–26. 10.1016/j.neurobiolaging.2016.09.005 28199888

[B8] Cao K. Du Y. Bao X. Han M. Su R. Pang J. (2022). Glutathione-bioimprinted nanoparticles targeting of N6-methyladenosine FTO demethylase as a strategy against leukemic stem cells. Small 18, e2106558. 10.1002/smll.202106558 35119204

[B9] Capelletti M. M. Manceau H. Puy H. Peoc'h K. (2020). Ferroptosis in liver diseases: an overview. Int. J. Mol. Sci. 21, 4908. 10.3390/ijms21144908 32664576 PMC7404091

[B10] Chen S. Xia H. Sheng L. (2023). WTAP-mediated m6A modification on circCMTM3 inhibits hepatocellular carcinoma ferroptosis by recruiting IGF2BP1 to increase PARK7 stability. Dig. Liver Dis. 55, 967–981. 10.1016/j.dld.2022.12.005 36586770

[B11] Chen X. Kang R. Kroemer G. Tang D. (2021a). Broadening horizons: the role of ferroptosis in cancer. Nat. Rev. Clin. Oncol. 18, 280–296. 10.1038/s41571-020-00462-0 33514910

[B12] Chen X. Li J. Kang R. Klionsky D. J. Tang D. (2021b). Ferroptosis: machinery and regulation. Autophagy 17, 2054–2081. 10.1080/15548627.2020.1810918 32804006 PMC8496712

[B13] Conrad M. Pratt D. A. (2019). The chemical basis of ferroptosis. Nat. Chem. Biol. 15, 1137–1147. 10.1038/s41589-019-0408-1 31740834

[B14] Cosialls E. El Hage R. Dos Santos L. Gong C. Mehrpour M. Hamai A. (2021). Ferroptosis: cancer stem cells rely on iron until "to die for" it. Cells 10, 2981. 10.3390/cells10112981 34831207 PMC8616391

[B15] Diao H. Tan H. Hu Y. Wang R. Cai P. Huang B. (2023). The m(6)A reader YTHDF1 promotes lung carcinoma progression via regulating ferritin mediate ferroptosis in an m(6)a-dependent manner. Pharm. (Basel) 16, 185. 10.3390/ph16020185 PMC9966794 37259333

[B16] Dixon S. J. Lemberg K. M. Lamprecht M. R. Skouta R. Zaitsev E. M. Gleason C. E. (2012). Ferroptosis: an iron-dependent form of nonapoptotic cell death. Cell 149, 1060–1072. 10.1016/j.cell.2012.03.042 22632970 PMC3367386

[B17] Doll S. Conrad M. (2017). Iron and ferroptosis: a still ill-defined liaison. IUBMB Life 69, 423–434. 10.1002/iub.1616 28276141

[B18] Doll S. Proneth B. Tyurina Y. Y. Panzilius E. Kobayashi S. Ingold I. (2017). ACSL4 dictates ferroptosis sensitivity by shaping cellular lipid composition. Nat. Chem. Biol. 13, 91–98. 10.1038/nchembio.2239 27842070 PMC5610546

[B19] Du Y. Hou G. Zhang H. Dou J. He J. Guo Y. (2018). SUMOylation of the m6A-RNA methyltransferase METTL3 modulates its function. Nucleic Acids Res. 46, 5195–5208. 10.1093/nar/gky156 29506078 PMC6007514

[B20] Fan Z. Yang G. Zhang W. Liu Q. Liu G. Liu P. (2021). Hypoxia blocks ferroptosis of hepatocellular carcinoma via suppression of METTL14 triggered YTHDF2-dependent silencing of SLC7A11. J. Cell Mol. Med. 25, 10197–10212. 10.1111/jcmm.16957 34609072 PMC8572766

[B21] Fu Y. Dominissini D. Rechavi G. He C. (2014). Gene expression regulation mediated through reversible m⁶A RNA methylation. Nat. Rev. Genet. 15, 293–306. 10.1038/nrg3724 24662220

[B22] Gao M. Monian P. Quadri N. Ramasamy R. Jiang X. (2015). Glutaminolysis and transferrin regulate ferroptosis. Mol. Cell 59, 298–308. 10.1016/j.molcel.2015.06.011 26166707 PMC4506736

[B23] Gong Y. Luo G. Zhang S. Chen Y. Hu Y. (2024). Transcriptome sequencing analysis reveals miR-30c-5p promotes ferroptosis in cervical cancer and inhibits growth and metastasis of cervical cancer xenografts by targeting the METTL3/KRAS axis. Cell Signal 111068, 111068. 10.1016/j.cellsig.2024.111068 38286198

[B24] Guo X. Liu F. Deng J. Dai P. Qin Y. Li Z. (2020). Electron-accepting micelles deplete reduced nicotinamide adenine Dinucleotide phosphate and impair two antioxidant cascades for ferroptosis-induced tumor eradication. ACS Nano 14, 14715–14730. 10.1021/acsnano.0c00764 33156626

[B25] Habaxi K. Wang W. Taximaimaiti M. Wang L. (2024). Methylation regulation of LPCAT3 improves osteoarthritis by regulating ACSL4 to inhibit chondrocyte ferroptosis. Crit. Rev. Eukaryot. Gene Expr. 34, 77–86. 10.1615/CritRevEukaryotGeneExpr.2023049244 38073444

[B26] Hambright W. S. Fonseca R. S. Chen L. Na R. Ran Q. (2017). Ablation of ferroptosis regulator glutathione peroxidase 4 in forebrain neurons promotes cognitive impairment and neurodegeneration. Redox Biol. 12, 8–17. 10.1016/j.redox.2017.01.021 28212525 PMC5312549

[B27] Haussmann I. U. Bodi Z. Sanchez-Moran E. Mongan N. P. Archer N. Fray R. G. (2016). m(6)A potentiates Sxl alternative pre-mRNA splicing for robust Drosophila sex determination. Nature 540, 301–304. 10.1038/nature20577 27919081

[B28] Huang H. Weng H. Chen J. (2020). The biogenesis and precise control of RNA m(6)A methylation. Trends Genet. 36, 44–52. 10.1016/j.tig.2019.10.011 31810533 PMC6925345

[B29] Huang H. Weng H. Sun W. Qin X. Shi H. Wu H. (2018). Recognition of RNA N(6)-methyladenosine by IGF2BP proteins enhances mRNA stability and translation. Nat. Cell Biol. 20, 285–295. 10.1038/s41556-018-0045-z 29476152 PMC5826585

[B30] Huang W. Qi C. B. Lv S. W. Xie M. Feng Y. Q. Huang W. H. (2016). Determination of DNA and RNA methylation in circulating tumor cells by mass spectrometry. Anal. Chem. 88, 1378–1384. 10.1021/acs.analchem.5b03962 26707930

[B31] Huang W. M. Li Z. X. Wu Y. H. Shi Z. L. Mi J. L. Hu K. (2023). m6A demethylase FTO renders radioresistance of nasopharyngeal carcinoma via promoting OTUB1-mediated anti-ferroptosis. Transl. Oncol. 27, 101576. 10.1016/j.tranon.2022.101576 36343416 PMC9646990

[B32] Imai H. Matsuoka M. Kumagai T. Sakamoto T. Koumura T. (2017). Lipid peroxidation-dependent cell death regulated by GPx4 and ferroptosis. Curr. Top. Microbiol. Immunol. 403, 143–170. 10.1007/82_2016_508 28204974

[B33] Ji F. H. Fu X. H. Li G. Q. He Q. Qiu X. G. (2022). FTO prevents thyroid cancer progression by SLC7A11 m6A methylation in a ferroptosis-dependent manner. Front. Endocrinol. (Lausanne) 13, 857765. 10.3389/fendo.2022.857765 35721711 PMC9205202

[B34] Jia G. Fu Y. Zhao X. Dai Q. Zheng G. Yang Y. (2011). N6-methyladenosine in nuclear RNA is a major substrate of the obesity-associated FTO. Nat. Chem. Biol. 7, 885–887. 10.1038/nchembio.687 22002720 PMC3218240

[B35] Jiang L. Kon N. Li T. Wang S. J. Su T. Hibshoosh H. (2015). Ferroptosis as a p53-mediated activity during tumour suppression. Nature 520, 57–62. 10.1038/nature14344 25799988 PMC4455927

[B36] Jiang X. Stockwell B. R. Conrad M. (2021). Ferroptosis: mechanisms, biology and role in disease. Nat. Rev. Mol. Cell Biol. 22, 266–282. 10.1038/s41580-020-00324-8 33495651 PMC8142022

[B37] Jin Y. Qiu J. Lu X. Ma Y. Li G. (2023). LncRNA CACNA1G-AS1 up-regulates FTH1 to inhibit ferroptosis and promote malignant phenotypes in ovarian cancer cells. Oncol. Res. 31, 169–179. 10.32604/or.2023.027815 37304234 PMC10208029

[B38] Jung M. Mertens C. Tomat E. Brune B. (2019). Iron as a central player and promising target in cancer progression. Int. J. Mol. Sci. 20, 273. 10.3390/ijms20020273 30641920 PMC6359419

[B39] Li J. Zou C. Zhang Z. Xue F. (2023a). N(6)-methyladenosine (m(6)A) reader YTHDF2 accelerates endothelial cells ferroptosis in cerebrovascular atherosclerosis. Mol. Cell Biochem. 10.1007/s11010-023-04858-1 37792239

[B40] Li W. Huang G. Wei J. Cao H. Jiang G. (2023b). ALKBH5 inhibits thyroid cancer progression by promoting ferroptosis through TIAM1-Nrf2/HO-1 axis. Mol. Cell Biochem. 478, 729–741. 10.1007/s11010-022-04541-x 36070054

[B41] Lin S. Choe J. Du P. Triboulet R. Gregory R. I. (2016). The m(6)A methyltransferase METTL3 promotes translation in human cancer cells. Mol. Cell 62, 335–345. 10.1016/j.molcel.2016.03.021 27117702 PMC4860043

[B42] Lin Y. Shen X. Ke Y. Lan C. Chen X. Liang B. (2022). Activation of osteoblast ferroptosis via the METTL3/ASK1-p38 signaling pathway in high glucose and high fat (HGHF)-induced diabetic bone loss. FASEB J. 36, e22147. 10.1096/fj.202101610R 35104016

[B43] Liu J. Dou X. Chen C. Chen C. Liu C. Xu M. M. (2020). N (6)-methyladenosine of chromosome-associated regulatory RNA regulates chromatin state and transcription. Science 367, 580–586. 10.1126/science.aay6018 31949099 PMC7213019

[B44] Liu J. Yue Y. Han D. Wang X. Fu Y. Zhang L. (2014). A METTL3-METTL14 complex mediates mammalian nuclear RNA N6-adenosine methylation. Nat. Chem. Biol. 10, 93–95. 10.1038/nchembio.1432 24316715 PMC3911877

[B45] Liu L. He J. Sun G. Huang N. Bian Z. Xu C. (2022a). The N6-methyladenosine modification enhances ferroptosis resistance through inhibiting SLC7A11 mRNA deadenylation in hepatoblastoma. Clin. Transl. Med. 12, e778. 10.1002/ctm2.778 35522946 PMC9076012

[B46] Liu L. Yao H. Zhou X. Chen J. Chen G. Shi X. (2022b). MiR-15a-3p regulates ferroptosis via targeting glutathione peroxidase GPX4 in colorectal cancer. Mol. Carcinog. 61, 301–310. 10.1002/mc.23367 34727409

[B47] Luo J. Yu H. Yuan Z. Ye T. Hu B. (2023). ALKBH5 decreases SLC7A11 expression by erasing m6A modification and promotes the ferroptosis of colorectal cancer cells. Clin. Transl. Oncol. 25, 2265–2276. 10.1007/s12094-023-03116-6 36820954

[B48] Lv D. Zhong C. Dixit D. Yang K. Wu Q. Godugu B. (2023). EGFR promotes ALKBH5 nuclear retention to attenuate N6-methyladenosine and protect against ferroptosis in glioblastoma. Mol. Cell 83, 4334–4351.e7. 10.1016/j.molcel.2023.10.025 37979586 PMC10842222

[B49] Ma L. Chen T. Zhang X. Miao Y. Tian X. Yu K. (2021a). The m(6)A reader YTHDC2 inhibits lung adenocarcinoma tumorigenesis by suppressing SLC7A11-dependent antioxidant function. Redox Biol. 38, 101801. 10.1016/j.redox.2020.101801 33232910 PMC7691619

[B50] Ma L. Zhang X. Yu K. Xu X. Chen T. Shi Y. (2021b). Targeting SLC3A2 subunit of system XC(-) is essential for m(6)A reader YTHDC2 to be an endogenous ferroptosis inducer in lung adenocarcinoma. Free Radic. Biol. Med. 168, 25–43. 10.1016/j.freeradbiomed.2021.03.023 33785413

[B51] Pang P. Si W. Wu H. Ju J. Liu K. Wang C. (2023). YTHDF2 promotes cardiac ferroptosis via degradation of SLC7A11 in cardiac ischemia-reperfusion injury. Antioxid. Redox Signal. 10.1089/ars.2023.0291 37548549

[B52] Pang P. Zhang S. Fan X. Zhang S. (2024). Knockdown of fat mass and obesity alleviates the ferroptosis in Parkinson's disease through m6A-NRF2-dependent manner. Cell Biol. Int. 10.1002/cbin.12118 38180302

[B53] Patil D. P. Chen C. K. Pickering B. F. Chow A. Jackson C. Guttman M. (2016). m(6)A RNA methylation promotes XIST-mediated transcriptional repression. Nature 537, 369–373. 10.1038/nature19342 27602518 PMC5509218

[B54] Qing Y. Dong L. Gao L. Li C. Li Y. Han L. (2021). R-2-hydroxyglutarate attenuates aerobic glycolysis in leukemia by targeting the FTO/m(6)A/PFKP/LDHB axis. Mol. Cell 81, 922–939.e9. 10.1016/j.molcel.2020.12.026 33434505 PMC7935770

[B55] Qiu M. L. Yan W. Liu M. M. (2023). Klf6 aggravates myocardial ischemia/reperfusion injury by activating Acsl4-mediated ferroptosis. Kaohsiung J. Med. Sci. 39, 989–1001. 10.1002/kjm2.12733 37530646 PMC11895878

[B56] Ruan F. Zeng J. Yin H. Jiang S. Cao X. Zheng N. (2021). RNA m6A modification alteration by black phosphorus quantum dots regulates cell ferroptosis: implications for nanotoxicological assessment. Small Methods 5, e2001045. 10.1002/smtd.202001045 34927824

[B57] Shao L. Hu F. Xu R. Nie H. Zhang H. Zhang P. (2023). METTL14 regulates the m6A modification of TRAF6 to suppress mitochondrial dysfunction and ferroptosis in dopaminergic neurons via the cGAS-STING pathway. Curr. Mol. Med. 24, 110107. 10.2174/0115665240263859231018110107 37881068

[B58] Shen H. Geng Z. Nie X. Liu T. (2023). Erianin induces ferroptosis of renal cancer stem cells via promoting ALOX12/P53 mRNA N6-methyladenosine modification. J. Cancer 14, 367–378. 10.7150/jca.81027 36860916 PMC9969579

[B59] Shen M. Guo M. Li Y. Wang Y. Qiu Y. Shao J. (2022). m(6)A methylation is required for dihydroartemisinin to alleviate liver fibrosis by inducing ferroptosis in hepatic stellate cells. Free Radic. Biol. Med. 182, 246–259. 10.1016/j.freeradbiomed.2022.02.028 35248719

[B60] Shen M. Li Y. Wang Y. Shao J. Zhang F. Yin G. (2021). N(6)-methyladenosine modification regulates ferroptosis through autophagy signaling pathway in hepatic stellate cells. Redox Biol. 47, 102151. 10.1016/j.redox.2021.102151 34607160 PMC8495178

[B61] Shriwas O. Priyadarshini M. Samal S. K. Rath R. Panda S. Das Majumdar S. K. (2020). DDX3 modulates cisplatin resistance in OSCC through ALKBH5-mediated m(6)A-demethylation of FOXM1 and NANOG. Apoptosis 25, 233–246. 10.1007/s10495-020-01591-8 31974865

[B62] Song Z. Jia G. Ma P. Cang S. (2021). Exosomal miR-4443 promotes cisplatin resistance in non-small cell lung carcinoma by regulating FSP1 m6A modification-mediated ferroptosis. Life Sci. 276, 119399. 10.1016/j.lfs.2021.119399 33781830

[B63] Stockwell B. R. Friedmann Angeli J. P. Bayir H. Bush A. I. Conrad M. Dixon S. J. (2017). Ferroptosis: a regulated cell death nexus linking metabolism, redox biology, and disease. Cell 171, 273–285. 10.1016/j.cell.2017.09.021 28985560 PMC5685180

[B64] Su R. Dong L. Li C. Nachtergaele S. Wunderlich M. Qing Y. (2018). R-2HG exhibits anti-tumor activity by targeting FTO/m(6)A/MYC/CEBPA signaling. Cell 172, 90–105. 10.1016/j.cell.2017.11.031 29249359 PMC5766423

[B65] Sun T. Wu R. Ming L. (2019). The role of m6A RNA methylation in cancer. Biomed. Pharmacother. 112, 108613. 10.1016/j.biopha.2019.108613 30784918

[B66] Sun X. Ou Z. Chen R. Niu X. Chen D. Kang R. (2016). Activation of the p62-Keap1-NRF2 pathway protects against ferroptosis in hepatocellular carcinoma cells. Hepatology 63, 173–184. 10.1002/hep.28251 26403645 PMC4688087

[B67] Tan M. He Y. Yi J. Chen J. Guo Q. Liao N. (2023). WTAP mediates NUPR1 regulation of LCN2 through m(6)A modification to influence ferroptosis, thereby promoting breast cancer proliferation, migration and invasion. Biochem. Genet. 10.1007/s10528-023-10423-8 37477758

[B68] Ueda Y. Ooshio I. Fusamae Y. Kitae K. Kawaguchi M. Jingushi K. (2017). AlkB homolog 3-mediated tRNA demethylation promotes protein synthesis in cancer cells. Sci. Rep. 7, 42271. 10.1038/srep42271 28205560 PMC5304225

[B69] Ursini F. Maiorino M. (2020). Lipid peroxidation and ferroptosis: the role of GSH and GPx4. Free Radic. Biol. Med. 152, 175–185. 10.1016/j.freeradbiomed.2020.02.027 32165281

[B70] Van Tran N. Ernst F. G. M. Hawley B. R. Zorbas C. Ulryck N. Hackert P. (2019). The human 18S rRNA m6A methyltransferase METTL5 is stabilized by TRMT112. Nucleic Acids Res. 47, 7719–7733. 10.1093/nar/gkz619 31328227 PMC6735865

[B71] Wang F. Sun Z. Zhang Q. Yang H. Yang G. Yang Q. (2023a). Curdione induces ferroptosis mediated by m6A methylation via METTL14 and YTHDF2 in colorectal cancer. Chin. Med. 18, 122. 10.1186/s13020-023-00820-x 37735401 PMC10512537

[B72] Wang H. Chen W. Cui Y. Gong H. Li H. (2023b). KIAA1429 protects hepatocellular carcinoma cells from ferroptotic cell death with a m(6) A-dependent posttranscriptional modification of SLC7A11. J. Cell Mol. Med. 27, 4118–4132. 10.1111/jcmm.17997 37830241 PMC10746954

[B73] Wang K. Wang G. Li G. Zhang W. Wang Y. Lin X. (2023c). m6A writer WTAP targets NRF2 to accelerate bladder cancer malignancy via m6A-dependent ferroptosis regulation. Apoptosis 28, 627–638. 10.1007/s10495-023-01817-5 36719469

[B74] Wang M. Mao C. Ouyang L. Liu Y. Lai W. Liu N. (2019). Long noncoding RNA LINC00336 inhibits ferroptosis in lung cancer by functioning as a competing endogenous RNA. Cell Death Differ. 26, 2329–2343. 10.1038/s41418-019-0304-y 30787392 PMC6889193

[B75] Wang P. Doxtader K. A. Nam Y. (2016). Structural basis for cooperative function of Mettl3 and Mettl14 methyltransferases. Mol. Cell 63, 306–317. 10.1016/j.molcel.2016.05.041 27373337 PMC4958592

[B76] Wang S. Wang Y. Li Q. Zeng K. Li X. Feng X. (2023d). RUNX1-IT1 favors breast cancer carcinogenesis through regulation of IGF2BP1/GPX4 axis. Discov. Oncol. 14, 42. 10.1007/s12672-023-00652-z 37036576 PMC10086083

[B77] Wang X. Huang N. Yang M. Wei D. Tai H. Han X. (2017). FTO is required for myogenesis by positively regulating mTOR-PGC-1α pathway-mediated mitochondria biogenesis. Cell Death Dis. 8, e2702. 10.1038/cddis.2017.122 28333151 PMC5386528

[B78] Wang X. Lu Z. Gomez A. Hon G. C. Yue Y. Han D. (2014). N6-methyladenosine-dependent regulation of messenger RNA stability. Nature 505, 117–120. 10.1038/nature12730 24284625 PMC3877715

[B79] Wang Y. Wang C. Guan X. Ma Y. Zhang S. Li F. (2023e). PRMT3-Mediated arginine methylation of METTL14 promotes malignant progression and treatment resistance in endometrial carcinoma. Adv. Sci. (Weinh) 10, e2303812. 10.1002/advs.202303812 37973560 PMC10754120

[B80] Wang Y. Zhao G. Condello S. Huang H. Cardenas H. Tanner E. J. (2021). Frizzled-7 identifies platinum-tolerant ovarian cancer cells susceptible to ferroptosis. Cancer Res. 81, 384–399. 10.1158/0008-5472.CAN-20-1488 33172933 PMC7855035

[B81] Wang Z. Li H. Cai H. Liang J. Jiang Y. Song F. (2023f). FTO sensitizes oral squamous cell carcinoma to ferroptosis via suppressing ACSL3 and GPX4. Int. J. Mol. Sci. 24, 16339. 10.3390/ijms242216339 38003537 PMC10671523

[B82] Warda A. S. Kretschmer J. Hackert P. Lenz C. Urlaub H. Hobartner C. (2017). Human METTL16 is a N(6)-methyladenosine (m(6)A) methyltransferase that targets pre-mRNAs and various non-coding RNAs. EMBO Rep. 18, 2004–2014. 10.15252/embr.201744940 29051200 PMC5666602

[B83] Wu J. Minikes A. M. Gao M. Bian H. Li Y. Stockwell B. R. (2019a). Intercellular interaction dictates cancer cell ferroptosis via NF2-YAP signalling. Nature 572, 402–406. 10.1038/s41586-019-1426-6 31341276 PMC6697195

[B84] Wu R. Li A. Sun B. Sun J. G. Zhang J. Zhang T. (2019b). A novel m(6)A reader Prrc2a controls oligodendroglial specification and myelination. Cell Res. 29, 23–41. 10.1038/s41422-018-0113-8 30514900 PMC6318280

[B85] Wu Y. Li H. Huang Y. Chen Q. (2023). Silencing of m(6)A methyltransferase KIAA1429 suppresses the progression of non-small cell lung cancer by promoting the p53 signaling pathway and ferroptosis. Am. J. Cancer Res. 13, 5320–5333. 38058803 PMC10695787

[B86] Xiao W. Adhikari S. Dahal U. Chen Y. S. Hao Y. J. Sun B. F. (2016). Nuclear m(6)A reader YTHDC1 regulates mRNA splicing. Mol. Cell 61, 507–519. 10.1016/j.molcel.2016.01.012 26876937

[B87] Xu K. Yang Y. Feng G. H. Sun B. F. Chen J. Q. Li Y. F. (2017). Mettl3-mediated m(6)A regulates spermatogonial differentiation and meiosis initiation. Cell Res. 27, 1100–1114. 10.1038/cr.2017.100 28809392 PMC5587845

[B88] Xu Y. Lv D. Yan C. Su H. Zhang X. Shi Y. (2022). METTL3 promotes lung adenocarcinoma tumor growth and inhibits ferroptosis by stabilizing SLC7A11 m(6)A modification. Cancer Cell Int. 22, 11. 10.1186/s12935-021-02433-6 34996469 PMC8742440

[B89] Yamaguchi Y. Kasukabe T. Kumakura S. (2018). Piperlongumine rapidly induces the death of human pancreatic cancer cells mainly through the induction of ferroptosis. Int. J. Oncol. 52, 1011–1022. 10.3892/ijo.2018.4259 29393418

[B90] Yan H. F. Zou T. Tuo Q. Z. Xu S. Li H. Belaidi A. A. (2021). Ferroptosis: mechanisms and links with diseases. Signal Transduct. Target Ther. 6, 49. 10.1038/s41392-020-00428-9 33536413 PMC7858612

[B91] Yang H. Hu Y. Weng M. Liu X. Wan P. Hu Y. (2022). Hypoxia inducible lncRNA-CBSLR modulates ferroptosis through m6A-YTHDF2-dependent modulation of CBS in gastric cancer. J. Adv. Res. 37, 91–106. 10.1016/j.jare.2021.10.001 35499052 PMC9039740

[B92] Yang W. H. Ding C. C. Sun T. Rupprecht G. Lin C. C. Hsu D. (2019). The hippo pathway effector TAZ regulates ferroptosis in renal cell carcinoma. Cell Rep. 28, 2501–2508. 10.1016/j.celrep.2019.07.107 31484063 PMC10440760

[B93] Yang W. S. Kim K. J. Gaschler M. M. Patel M. Shchepinov M. S. Stockwell B. R. (2016). Peroxidation of polyunsaturated fatty acids by lipoxygenases drives ferroptosis. Proc. Natl. Acad. Sci. U. S. A. 113, E4966–E4975. 10.1073/pnas.1603244113 27506793 PMC5003261

[B94] Yang W. S. Sriramaratnam R. Welsch M. E. Shimada K. Skouta R. Viswanathan V. S. (2014). Regulation of ferroptotic cancer cell death by GPX4. Cell 156, 317–331. 10.1016/j.cell.2013.12.010 24439385 PMC4076414

[B95] Ye F. Wu J. Zhang F. (2023). METTL16 epigenetically enhances GPX4 expression via m6A modification to promote breast cancer progression by inhibiting ferroptosis. Biochem. Biophys. Res. Commun. 638, 1–6. 10.1016/j.bbrc.2022.10.065 36434904

[B96] Ye J. Chen X. Jiang X. Dong Z. Hu S. Xiao M. (2022). RNA demethylase ALKBH5 regulates hypopharyngeal squamous cell carcinoma ferroptosis by posttranscriptionally activating NFE2L2/NRF2 in an m(6) A-IGF2BP2-dependent manner. J. Clin. Lab. Anal. 36, e24514. 10.1002/jcla.24514 35689537 PMC9279968

[B97] Yu M. Gai C. Li Z. Ding D. Zheng J. Zhang W. (2019). Targeted exosome-encapsulated erastin induced ferroptosis in triple negative breast cancer cells. Cancer Sci. 110, 3173–3182. 10.1111/cas.14181 31464035 PMC6778638

[B98] Yuan S. Xi S. Weng H. Guo M. M. Zhang J. H. Yu Z. P. (2023). YTHDC1 as a tumor progression suppressor through modulating FSP1-dependent ferroptosis suppression in lung cancer. Cell Death Differ. 30, 2477–2490. 10.1038/s41418-023-01234-w 37903990 PMC10733405

[B99] Yue Y. Liu J. Cui X. Cao J. Luo G. Zhang Z. (2018). VIRMA mediates preferential m(6)A mRNA methylation in 3'UTR and near stop codon and associates with alternative polyadenylation. Cell Discov. 4, 10. 10.1038/s41421-018-0019-0 29507755 PMC5826926

[B100] Zhang C. Fu J. Zhou Y. (2019). A review in research progress concerning m6A methylation and immunoregulation. Front. Immunol. 10, 922. 10.3389/fimmu.2019.00922 31080453 PMC6497756

[B101] Zhang G. Mi W. Wang C. Li J. Zhang Y. Liu N. (2023a). Targeting AKT induced Ferroptosis through FTO/YTHDF2-dependent GPX4 m6A methylation up-regulating and degradating in colorectal cancer. Cell Death Discov. 9, 457. 10.1038/s41420-023-01746-x 38102129 PMC10724184

[B102] Zhang H. Deng T. Liu R. Ning T. Yang H. Liu D. (2020). CAF secreted miR-522 suppresses ferroptosis and promotes acquired chemo-resistance in gastric cancer. Mol. Cancer 19, 43. 10.1186/s12943-020-01168-8 32106859 PMC7045485

[B103] Zhang L. Wang X. Che W. Zhou S. Feng Y. (2023b). METTL3 silenced inhibited the ferroptosis development via regulating the TFRC levels in the Intracerebral hemorrhage progression. Brain Res. 1811, 148373. 10.1016/j.brainres.2023.148373 37105375

[B104] Zhang Y. Shi J. Liu X. Feng L. Gong Z. Koppula P. (2018). BAP1 links metabolic regulation of ferroptosis to tumour suppression. Nat. Cell Biol. 20, 1181–1192. 10.1038/s41556-018-0178-0 30202049 PMC6170713

[B105] Zhao Y. Chen Y. Jin M. Wang J. (2021). The crosstalk between m(6)A RNA methylation and other epigenetic regulators: a novel perspective in epigenetic remodeling. Theranostics 11, 4549–4566. 10.7150/thno.54967 33754077 PMC7977459

[B106] Zheng G. Dahl J. A. Niu Y. Fedorcsak P. Huang C. M. Li C. J. (2013). ALKBH5 is a mammalian RNA demethylase that impacts RNA metabolism and mouse fertility. Mol. Cell 49, 18–29. 10.1016/j.molcel.2012.10.015 23177736 PMC3646334

[B107] Zheng J. Zhang Q. Zhao Z. Qiu Y. Zhou Y. Wu Z. (2023). Epigenetically silenced lncRNA SNAI3-AS1 promotes ferroptosis in glioma via perturbing the m(6)A-dependent recognition of Nrf2 mRNA mediated by SND1. J. Exp. Clin. Cancer Res. 42, 127. 10.1186/s13046-023-02684-3 37202791 PMC10197824

[B108] Zhou K. I. Shi H. Lyu R. Wylder A. C. Matuszek Z. Pan J. N. (2019). Regulation of Co-transcriptional pre-mRNA splicing by m(6)A through the low-complexity protein hnRNPG. Mol. Cell 76, 70–81. 10.1016/j.molcel.2019.07.005 31445886 PMC6778029

[B109] Zhuang S. Ma Y. Zeng Y. Lu C. Yang F. Jiang N. (2021). METTL14 promotes doxorubicin-induced cardiomyocyte ferroptosis by regulating the KCNQ1OT1-miR-7-5p-TFRC axis. Cell Biol. Toxicol. 39, 1015–1035. 10.1007/s10565-021-09660-7 34648132

[B110] Zou Y. Zheng S. Xie X. Ye F. Hu X. Tian Z. (2022). N6-methyladenosine regulated FGFR4 attenuates ferroptotic cell death in recalcitrant HER2-positive breast cancer. Nat. Commun. 13, 2672. 10.1038/s41467-022-30217-7 35562334 PMC9106694

